# Schistosomes Enhance Plasminogen Activation: The Role of Tegumental Enolase

**DOI:** 10.1371/journal.ppat.1005335

**Published:** 2015-12-11

**Authors:** Barbara C. Figueiredo, Akram A. Da'dara, Sergio C. Oliveira, Patrick J. Skelly

**Affiliations:** 1 Department of Infectious Disease and Global Health, Cummings School of Veterinary Medicine, Tufts University, North Grafton, Massachusetts, United States of America; 2 Departamento de Bioquímica e Imunologia, Instituto de Ciências Biológicas, Universidade Federal de Minas Gerais, Belo Horizonte, Minas Gerais, Brazil; 3 Instituto Nacional de Ciência e Tecnologia em Doenças Tropicais (INCT-DT), Conselho Nacional de Desenvolvimento Científico e Tecnológico, Ministério de Ciência Tecnologia e Inovação Salvador, Bahia, Brazil; Uniformed Services University, UNITED STATES

## Abstract

*Schistosoma mansoni* is a blood fluke parasite that causes schistosomiasis, a debilitating disease of global public health importance. These relatively large parasites are able to survive prolonged periods in the human vasculature without inducing stable blood clots around them. We show here that the intravascular life stages (schistosomula and adult males and females) can all promote significant plasminogen (PLMG) activation in the presence of tissue plasminogen activator (tPA). This results in the generation of the potent fibrinolytic agent plasmin which could degrade blood clots forming around the worms *in vivo*. We demonstrate that *S*. *mansoni* enolase (*Sm*Eno) is a host-interactive tegumental enzyme that, in recombinant form, can bind PLMG and promote its activation. Like classical members of the enolase protein family, SmEno can catalyze the interconversion of 2-phospho-D-glycerate (2-PGA) and phosphoenolpyruvate (PEP). The enzyme has maximal activity at pH 7.5, requires Mg^2+^ for optimal activity and can be inhibited by NaF but not mefloquin. Suppressing expression of the *SmEno* gene significantly diminishes enolase mRNA levels, protein levels and surface enzyme activity but, surprisingly, does not affect the ability of the worms to promote PLMG activation. Thus, while *Sm*Eno can enhance PLMG activation, our analysis suggests that it is not the only contributor to the parasite’s ability to perform this function. We show that the worms possess several other PLMG-binding proteins in addition to *Sm*Eno and these may have a greater importance in schistosome-driven PLMG activation.

## Author Summary

Schistosomiasis affects more than 200 million people worldwide and causes up to 280,000 deaths per year. In terms of global mortality and morbidity, this disease is the most important human helminth infection. *Schistosoma mansoni* parasites can live for years within human blood vessels and seem to be refractory to intravascular thrombus formation. We hypothesize that the parasites are able to promote fibrinolysis in order to avoid firm clot formation around them. In this work, we characterize *S*. *mansoni* enolase (*Sm*Eno), an enzyme that is found both inside and at the surface of the intravascular worms, and we evaluate its involvement in plasminogen (PLMG) activation. We demonstrate that *Sm*Eno is highly expressed in schistosomula, the larval intravascular stages of *S*. *mansoni*, as well as adult worms and eggs. We confirm the localization of *Sm*Eno within the parasites and at the host/parasite interface. We demonstrate that tegumental *Sm*Eno is enzymatically functional and it is able to bind to and enhance the activation of human PLMG. Live parasites whose *Sm*Eno gene has been suppressed using RNAi display lower surface enolase activity, but their ability to activate PLMG is unchanged compared to control parasites. Thus, our data show that *Sm*Eno can contribute to PLMG activation, although it is not the only schistosome molecule responsible for this activity.

## Introduction

*Schistosoma mansoni* is one of the etiological agents of schistosomiasis, which is considered the most important human helminth disease in terms of global morbidity and mortality. [1,2]. Schistosomiasis, together with other parasitosis, belongs to the group of tropical neglected diseases afflicting preferentially low-income, developing countries [3]. Considering the number of people infected (>200 million) and those at risk of infection (~800 million), schistosomiasis ranks second only to malaria in importance among parasitic diseases [4]. The disease can cause abdominal pain, portal hypertension and hepatic and intestinal fibrosis in chronically infected patients. Schistosomiasis is a major public health problem in endemic countries [2,4].

When a person is infected, larval schistosomes (schistosomula) migrate to the blood vessels where they mature and live as pairs in the mesenteric or perivesicular veins for many years [5]. Although the parasites trigger immune responses, they appear to possess efficient mechanisms to evade immune-mediated damage [6]. The relatively large adult schistosome pair can disturb blood flow and this is a potential activator of blood coagulation [7]. However, blood clots are not observed around the parasites within the blood vessels of infected animals and experimental evidence demonstrates that platelets do not bind to schistosomes *in vivo* or *in vitro* [8,9].

Blood clots are composed of cellular material (largely platelets) as well as coagulation proteins (largely cross-linked fibrin). Several mechanisms have been proposed by which schistosomes both inhibit blood clot formation as well as promote the lysis of any blood clots that do manage to form in their vicinity (reviewed in [10]) Among the latter is the proposed ability of the parasites to hijack the hosts own system of clot dissolution by which fibrin is degraded proteolytically. Under normal conditions this process begins when the zymogen plasminogen (PLMG) is converted by e.g. tissue plasminogen activator (tPA) into its enzymatically active form, plasmin, which hydrolyses cross-linked fibrin to dissolve blood clots. It has been proposed that schistosomes can bind plasminogen and promote its conversion to plasmin to impede stable blood clot formation [11]. A variety of other pathogenic organisms, including bacteria [12], fungi [13], and protozoa [14,15] also possess the ability to interact with plasminogen. In the case of *Schistosoma bovis*, plasminogen has been shown to bind directly to the surface of adult male parasites (but not to females) [11]. Additionally, it has been shown that *S*. *bovis* tegumental extracts contain several plasminogen-binding proteins; among the most prominent is the enzyme enolase [11]. Enolase is best known as a key enzyme in intracellular glucose metabolism [16,17]. In recent years the protein has also been identified on the surface of the cells of a variety of organisms (despite the fact that it is not predicted to possess any transmembrane domains or signal peptides that might facilitate its conventional release to the exterior) [18]. Enolase has been described on the tegument of *S*. *bovis* [19] and *S*. *japonicum* [20] and has been identified in proteomic analysis of the tegument of *S*. *mansoni* [21–23]. In this study we monitor the ability of *S*. *mansoni* parasites to activate PLMG and we characterize *S*. *mansoni* enolase (*Sm*Eno) in order to determine its contribution to PLMG activation at the host-parasite interface.

## Results

### *Schistosoma mansoni* parasites activate plasminogen

**Fig 1:**
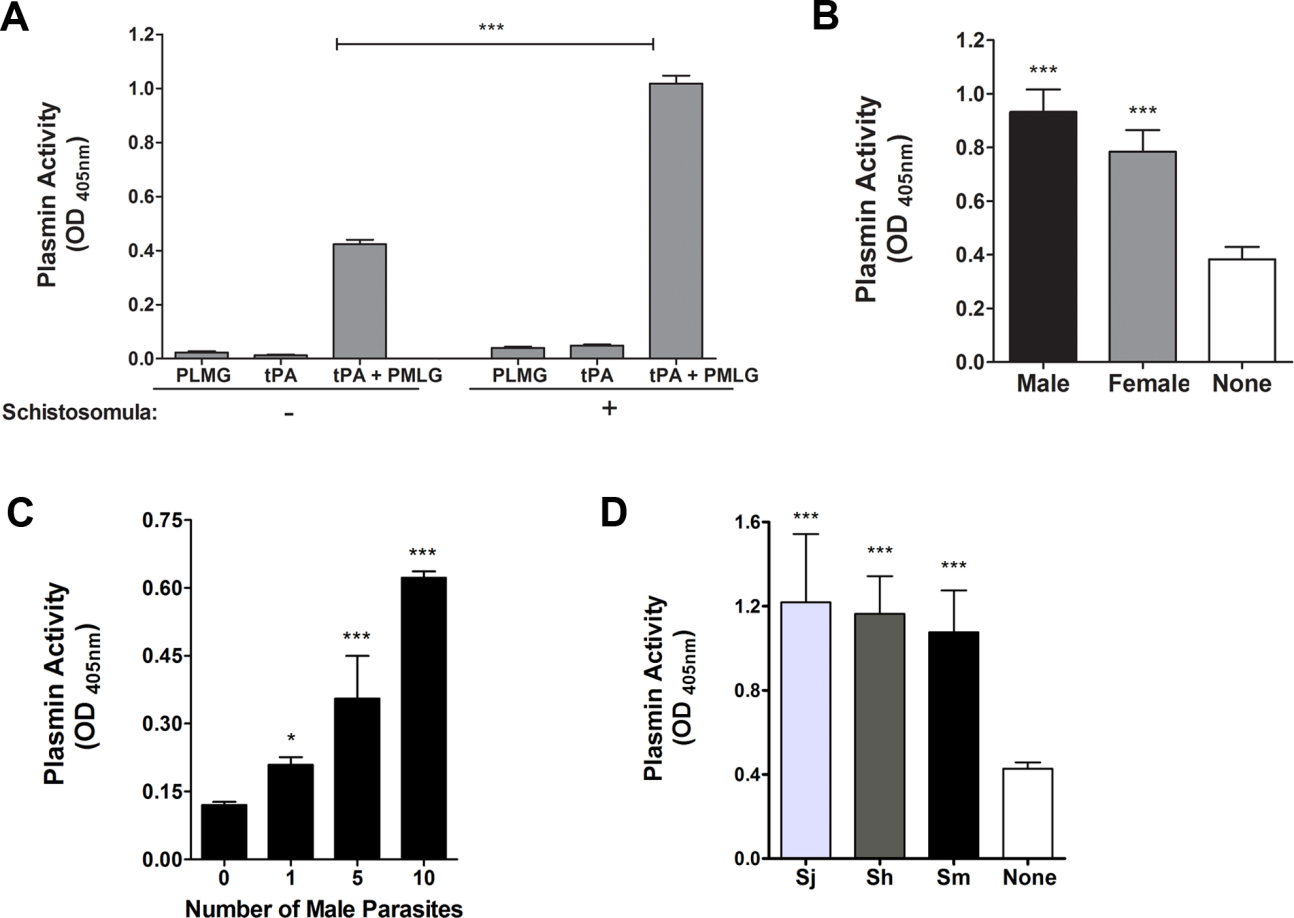
Plasminogen (PLMG) activation by schistosomes in the presence of tissue plasminogen activator (tPA). (**A**) Plasmin activity (mean OD_405_ value +/- SD; 60 min) detected in the presence (+) or absence (-) of live schistosomula (1000 parasites per well, n≥5). (**B**) Plasmin activity (mean OD_405_ value +/- SD; 60 min) detected in the absence (white bar) or presence of male (black bar) or female (gray bar) adult schistosomes. In each case, ≥5 adult worms were evaluated individually. (**C**) Plasmin activity (mean OD_405_ value +/- SD; 30 min) detected in the presence of different numbers of male parasites as indicated, n≥5. (**D**) Plasmin activity (mean OD_405_ value +/- SD; 60 min) detected in the absence (white bar) or presence of individual male *S*. *japonicum* (Sj), *S*. *haematobium* (Sh) or *S*. *mansoni* (Sm), n ≥10. Significant differences from control conditions (reagents themselves without parasites) are denoted by *, p<0.05, and ***, p <0.001.

Plasminogen (PLMG) can be activated *in vivo* by tissue plasminogen activator (tPA) to generate plasmin. As shown in Fig 1A, this plasmin can cleave the synthetic substrate D-Valyl-L-Leucyl-L-Lysine 4-nitroanilide dihydrochloride to generate product that is detected at OD_405_ (lane “tPA + PLMG” in the minus schistosomula group, left). As expected, plasminogen or tPA alone exhibit negligible activity in this assay (lanes PLMG and tPA). Adding schistosomula to the assay has no significant impact on the outcome when only PLMG or tPA are present but dramatically enhances plasminogen activation in the presence of tPA (lane “tPA + PLMG” plus schistosomula, right). Fig 1B shows that adult schistosomes (both males and females) also promote plasminogen activation in the presence of tPA. Fig 1C shows that the worms exert this effect in a dose-dependent manner: the more parasites added the greater that activation. This phenomenon is not confined to *S*. *mansoni*; as illustrated in Fig 1D, adult male *S*. *japonicum* and *S*. *haematobium* also promote plasminogen activation and to a comparable degree.

### *Schistosoma mansoni* enolase

Members of the enolase protein family have been shown to be involved in PLMG activation in other systems and, in *S*. *mansoni*, an enolase homolog has been identified by proteomics in the tegument where it might interact with host plasminogen. To investigate, we first cloned the cDNA encoding *S*. *mansoni* enolase (*Sm*Eno) in this work. This cDNA potentially encodes a 434-amino acid protein, predicted to be a soluble 47-kDa polypeptide with a pI of 6.2. (GenBank accession number: Q27877). *Sm*Eno has the enolase signature motif spanning residues 341–354 (^341^LLLKVNQIGSLTES^354^) as well as five highly conserved amino acids reported to be essential for substrate stabilization at H^158^, E^167^, E^210^, K^344^ and K^395^ and four conserved Mg^2+^ binding amino acids: D^245^, E^294^, D^319^ and K^395^. Twelve potential phosphorylation sites are predicted (S^14^, T^26^, T^41^, T^52^, Y^57^, S^177^, Y^189^, T^237^, S^266^, S^292^, S^354^ and S^374^) while no signal peptide, transmembrane domains or O-glycosylation sites are predicted. The ^296^FDQDDWGAW^304^ motif exhibits 7/9 residue identity with a conserved caveolin-binding domain [24]. *Sm*Eno also possesses several conserved amino acid residues that are important for PLMG-binding (both at an internal motif ^251^FHKNGKY^257^ and also at the C-terminus—the penultimate residue is K^433^) suggesting that *Sm*Eno can bind PLMG [25,26].

### *Sm*Eno is highly expressed in intravascular life stages

**Fig 2:**
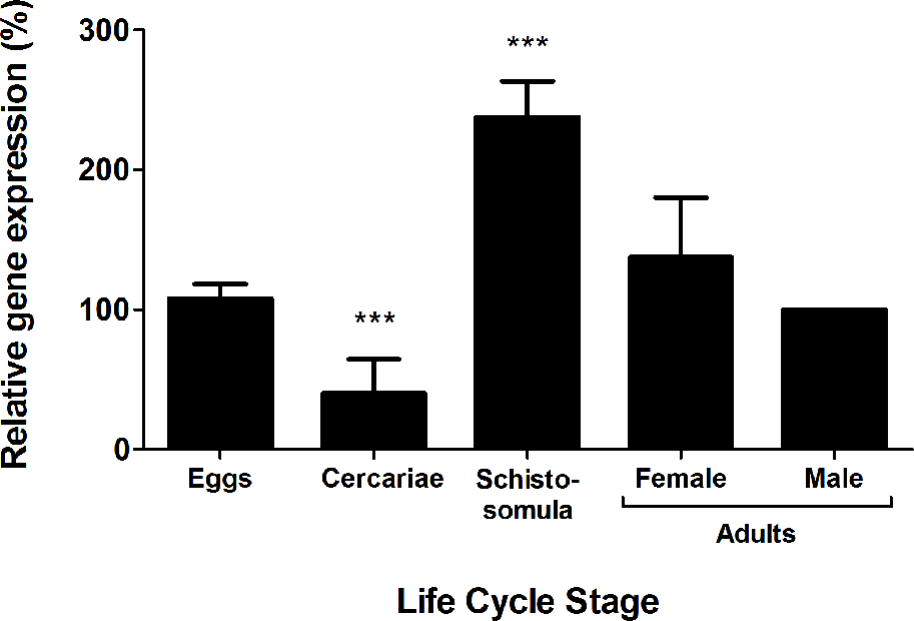
Expression profile of *SmEno* at different stages in the *S*. *mansoni* life cycle. Quantitative RT-PCR data showing relative expression level (mean +/- SD) of *SmEno* at different stages in the *S*. *mansoni* life cycle: eggs, cercariae, schistosomula (7-day cultured larvae), adult female worms, and adult males (set at 100%). Results are representative of two independent experiments. Significant differences between male adult worms and other life stages is denoted by ***, p <0.001.

The relative expression of *SmEno* was evaluated in different stages in the life cycle of *S*. *mansoni* by quantitative real-time PCR (qRT-PCR) and results are presented in Fig 2. The *SmEno* gene exhibited high relative expression in the intravascular life stages; with highest levels seen in schistosomula (7-day old). Lowest relative expression was detected in cercariae.

### The *S*. *mansoni* tegument external surface has a functional enolase

#### Immunolocalization

**Fig 3:**
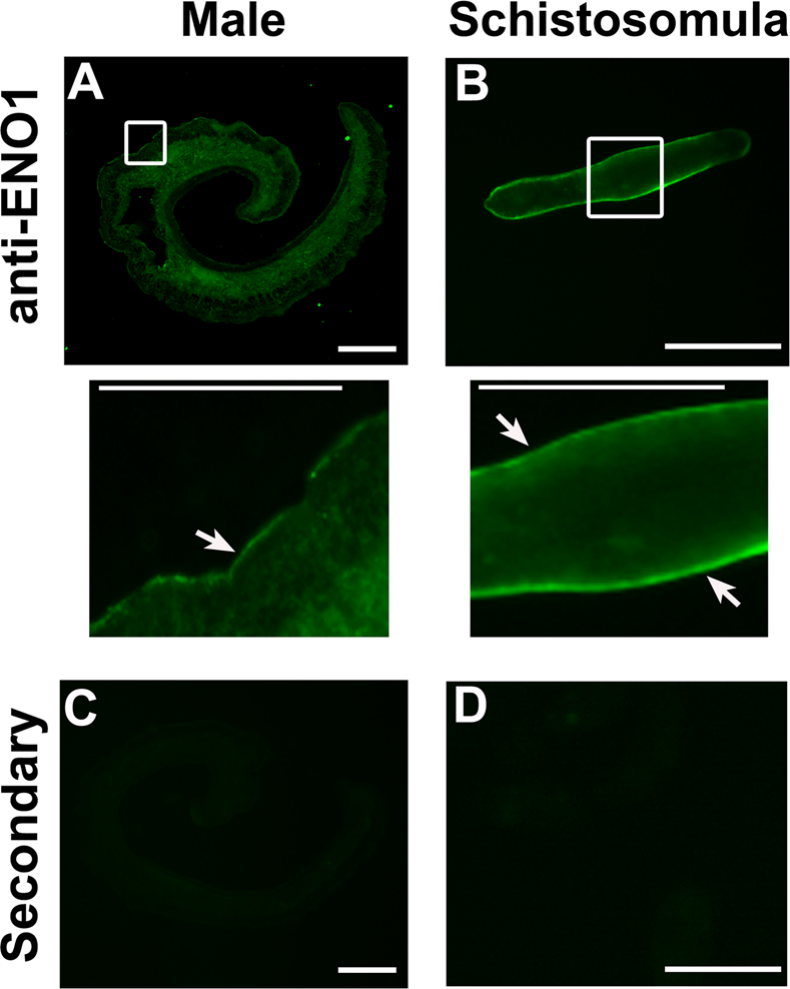
Immunolocalization of *Sm*Eno in *S*. *mansoni* adult worms and schistosomula. Indirect immunofluorescent labeling of native *Sm*Eno protein in sections of (**A**) an adult male and (**B**) a whole fixed schistosomulum using polyclonal anti-ENO1 antibody (and secondary anti-rabbit IgG antibody conjugated to Alexa 488 (green)). Enlargements of the areas shown in white boxes in the top row are presented in the middle row. Arrows indicate clear tegumental staining. As a control, secondary antibody alone was used on sections of **(C)** adult males and **(D**), whole fixed schistosomula. Scale bars = 100 μm or 50 μm in insets (middle row).

To localize *Sm*Eno, sections of adult male parasites and whole 7-day cultured schistosomula were stained with polyclonal anti-ENO1 antibodies (Sigma-Aldrich, Saint Louis, USA). *Sm*Eno was located widely throughout the bodies of the adults (Fig 3A). The areas within the white boxes in A-B are enlarged in the middle row and these enlargements clearly show *Sm*Eno to be also located in the tegument (Fig 3, arrows). Tegumental staining is detected starkly in the paraformaldehyde-fixed schistosomula sample (Fig 3B, arrows). Since paraformaldehyde fixation precludes antibody entry into the schistosomula, this likely explains why no internal staining is seen here. Control parasites, exposed to secondary antibody alone, do not display signal in the tegument or elsewhere in either adult parasites or schistosomula (Fig 3C and 3D).

#### Enolase enzyme activity assay

**Fig 4:**
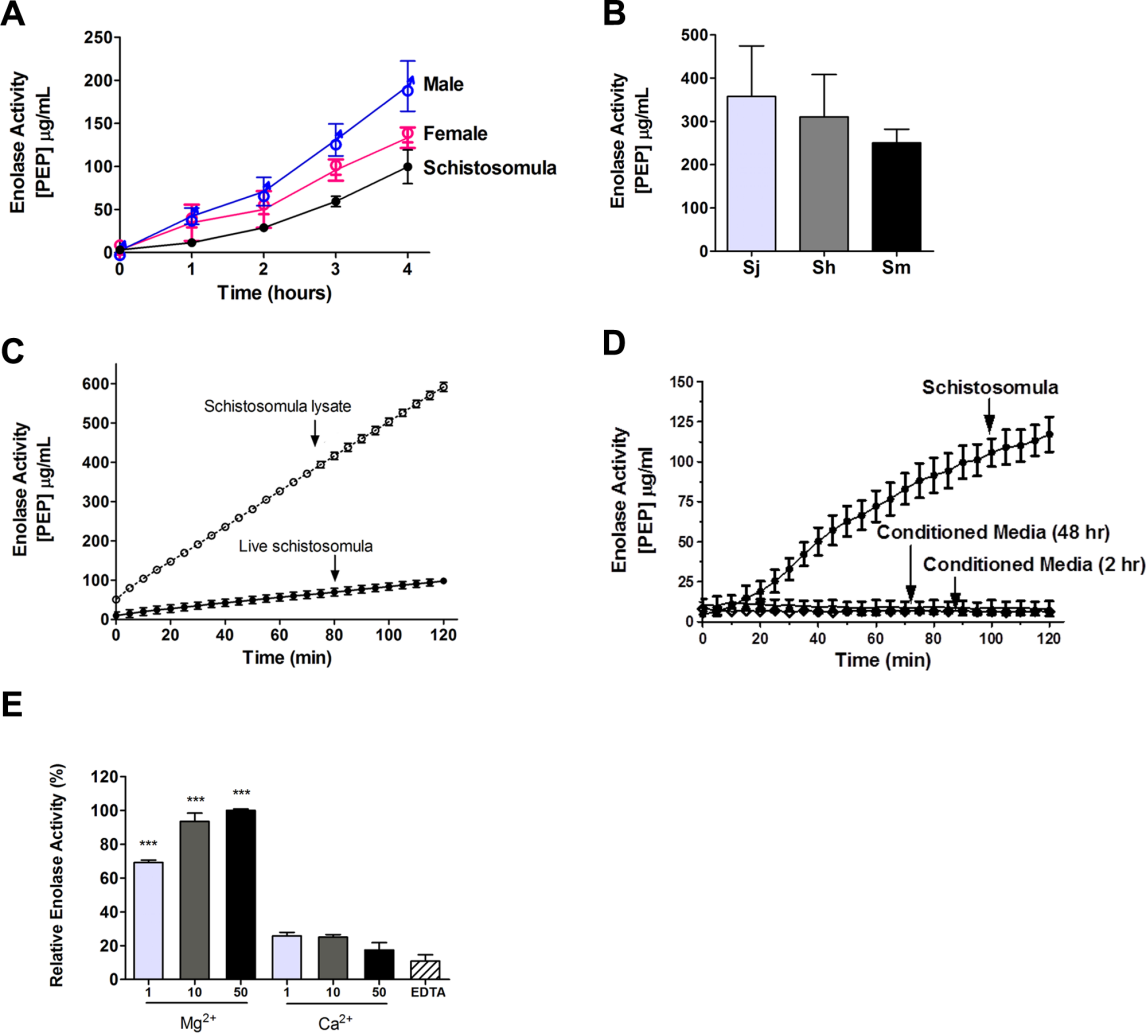
Schistosome enolase activity (mean PEP generated (μg/ml) +/- SD). (**A**) Enolase activity exhibited by live adult male or adult female worms (individuals) or schistosomula (~1000 parasites/sample) (n ≥ 10 replicates/sample). (**B**) Enolase activity exhibited by live *S*. *japonicum* (Sj), *S*. *haematobium* (Sh) and *S*. *mansoni* (Sm) adult males after 4 hours, n ≥ 12 replicates/sample. (**C**) Enolase activity exhibited by live schistosomula (~1,000/sample, black squares) versus total schistosomula lysate (open squares) over 2 hours, n ≥ 5/condition. (**D**) Enolase activity exhibited by live schistosomula (~1,000/sample, black circles) versus 2 h (open diamond) or 48 h (closed diamond), conditioned medium, n ≥ 5/condition. (**E**) Effect of varying divalent ion (Mg^2+^ or Ca^2+^) concentration (1, 10 or 50 mM as indicated) on mean relative enolase activity. The highest activity value (in 50 mM Mg^2+^) was set at 100% and activities relative to this are presented (n ≥ 5/condition). Significant differences relative to equivalent measurements containing Ca^2+^ are denoted by *** for p <0.001. EDTA is Ethylenediaminetetraacetic acid.

Enzyme activity assays were undertaken using live adult male parasites, adult female parasites and schistosomula, to monitor surface enolase activity in these life stages. The assay measures the generation of phosphoenolpyruvate (PEP, detected at OD_240_) from the added enolase substrate 2-phospho-D-glycerate (2-PGA). Adults were tested individually and schistosomula were tested in groups of ~1,000. As shown in Fig 4A, all *S*. *mansoni* life stages tested showed clear enolase activity. Individual males exhibit a generally higher level of enolase activity compared to females. Live, single male worms of the three major schistosome species that infect humans: *S*. *mansoni*, *S*. *haematobium* and *S*. *japonicum* were compared for surface enolase activity and Fig 4B shows the results of this analysis. It is clear that all three species exhibit clear enolase activity, with no significant difference in activity between them.

The enolase activity exhibited by live *S*. *mansoni* schistosomula was compared with that shown by a total parasite protein lysate (made from equivalent numbers of schistosomula). Not surprisingly, the activity exhibited by the lysate (containing intracellular as well as surface enolase) is considerably higher than that of the living schistosomula (which represents surface enolase alone). After two hours, mean tegumental enolase activity, was 18.5% that of the total lysate (Fig 4C).

Enolase activity was measured in culture medium that previously contained parasites in order to determine if the enzyme was being secreted by the worms or if it was leaking from the intracellular environment because of damage to the worms in culture. Parasites were first incubated in medium for 2 hours (to generate 2 h conditioned medium) and were then placed in fresh medium; enolase activity was monitored in both circumstances–in the 2 h conditioned medium and in the fresh medium containing worms. As demonstrated in Fig 4D, enolase activity was detected only in the sample containing worms. Furthermore, in medium that contained worms for 48 hours (48 h conditioned medium) again no enolase activity was detected. These data show that, even after prolonged (48 h) culture, no secretion or no damage that led to the leakage of internal enolase was evident.

Since enolases are metalloenzymes, that require the metal ion magnesium to be active, we compared tegumental enolase activity in live parasites in the presence of magnesium (Mg^2+^) versus calcium (Ca^2+^). Experiments using live schistosomula demonstrated high activity only in the presence of Mg^2+^. Ca^2+^ was unable to substitute for Mg^2+^ in this assay. Lowest activity was seen in the presence of the divalent cation chelator, EDTA (Fig 4E).

### Heterologous expression, purification and characterization of recombinant *Sm*Eno (r*Sm*Eno)

**Fig 5:**
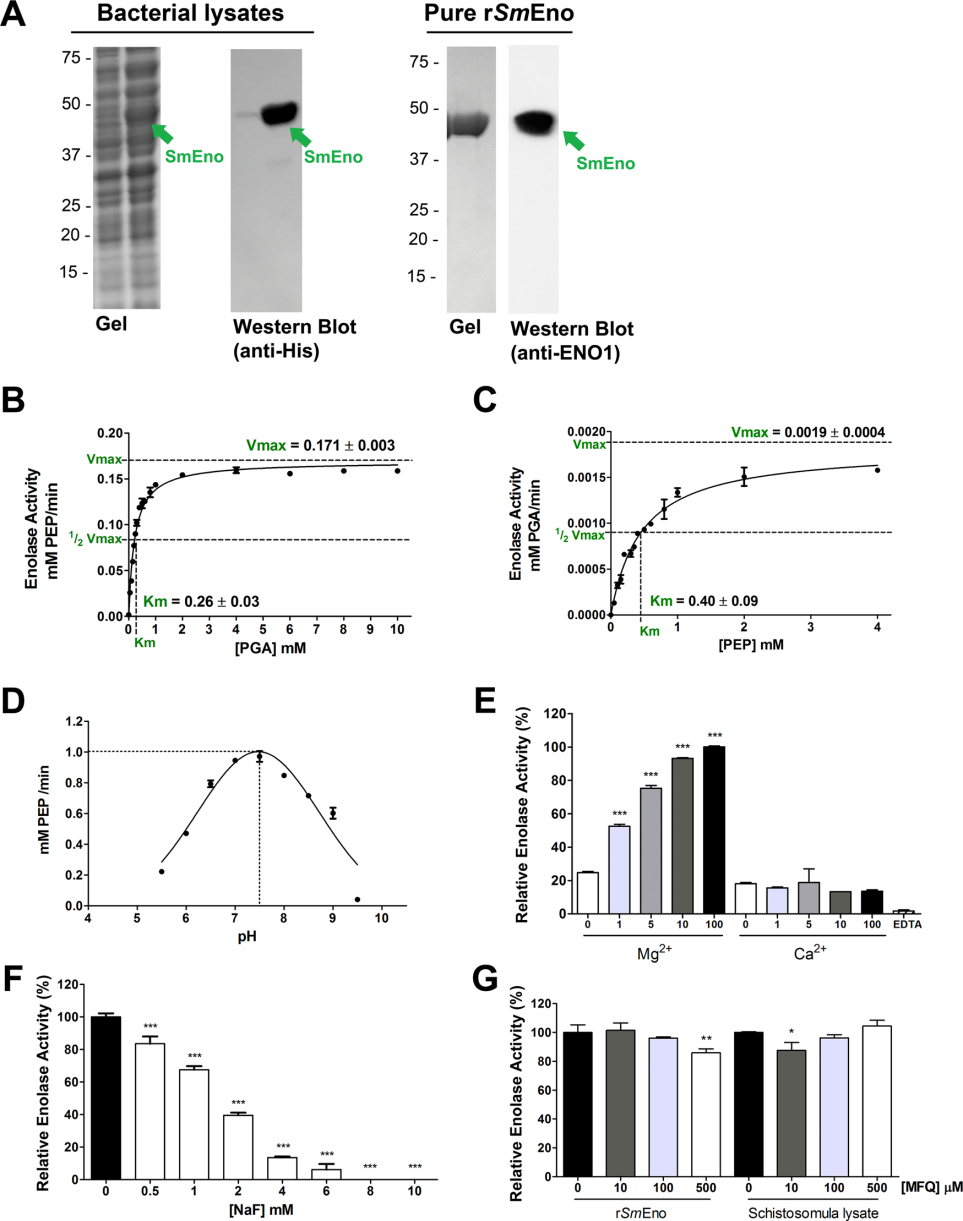
Heterologous expression, purification and kinetics of r*Sm*Eno. (**A**) Heterologous expression of r*Sm*Eno in *E*. *coli* BL21 Star (DE3). Coomassie-stained gel showing SDS-PAGE resolution of lysates of *E*. *coli* bacteria harboring pTrcHisB::*Sm*Eno before (Gel, left lane) or 4 hours after (Gel, right lane) protein expression induction. The arrow indicates r*Sm*Eno in the induced lane. In western blot analysis of these lysates probed with monoclonal anti-His tag antibody, a prominent ~50 kDa protein (*Sm*Eno) is detected (Western Blot, anti-His, arrow). Recombinant *Sm*Eno protein was purified from bacterial lysate by immobilized metal affinity chromatography. A single ~50 kDa pure protein (*Sm*Eno) is resolved following Coomassie brilliant blue staining of an SDS-PAGE gel and this protein binds anti-ENO antibody, as determined by western blot analysis (right lane, arrow). Positions of migration of molecular mass markers are indicated on the left (kDa). Michaelis-Menten kinetic curve generated using PGA as substrate (**B**, catalyzing the forward reaction) or using PEP as substrate (**C**, catalyzing the reverse reaction). The apparent Km and Vmax values shown represent the mean +/- SD of three independent experiments. (**D**) Recombinant *Sm*Eno activity in a buffer system covering the pH range 5.5–9.5. Enzymatic activity is maximal at pH 7.5. (**E**) Impact of divalent ion (Mg^2+^ or Ca^2+^, as indicated) concentration on mean r*Sm*Eno activity (± SD). The highest activity value (at 100 mM Mg^2+^) was set at 100% and relative activities were calculated and are presented. Significant differences relative to equivalent measurements containing Ca^2+^ are denoted by *** for p <0.001. EDTA is Ethylenediaminetetraacetic acid. (**F**) r*Sm*Eno activity in the presence of increasing concentrations of NaF (white bars) compared to its activity in the absence of inhibitors (set at 100%, black bar). Significant differences relative to the untreated control are denoted by *** for p <0.001. (**G**) Influence of increasing concentrations of mefloquine (MFQ) on r*Sm*Eno activity (left bars) or on schistosomula lysate enolase activity (right bars). Activity measured in the absence of MFQ was set at 100% and relative activities were calculated. Significant differences relative to the untreated control are denoted by * for p<0.05 and ** for p<0.01. In E-F, bars represent mean relative activity ± SD, n = 3.

The *SmEno* coding DNA was cloned into the pTrcHisB plasmid in frame with a hexa-His-tag domain as described in Methods. Heterologous expression was induced in *E*. *coli* and bacterial extracts were analyzed by SDS-PAGE followed by Coomassie blue staining. A prominent band of the approximate expected size of r*Sm*Eno (~50 kDa) was observed in the induced cell lysate (Fig 5A, left gel, arrow). Bacterial extracts were subjected to western blot analysis using a mouse monoclonal anti-His-tag antibody. This analysis confirmed that the induced protein of around 50 kDa had a histidine tag (Fig 5A, Western Blot, anti-His). The soluble His-tagged r*Sm*Eno protein was purified to homogeneity from bacterial lysates using nickel affinity chromatography (Fig 5A, right gel) and was detected by the anti-ENO1 antibody (Fig 5A, right Western Blot, anti-ENO1, arrow).

Purified r*Sm*Eno was tested to determine if it exhibited classical enolase activity—that is the ability to convert 2-PGA to PEP as well as to catalyze the reverse reaction (PEP to 2-PGA). This analysis confirmed *Sm*Eno to be a *bone fide* enolase. The kinetics of the forward reaction (with 2-PGA as substrate) are depicted in Fig 5B which reveal the Michaelis constant (Km) to be 0.26±0.03 mM and maximum velocity (Vmax) to be 0.171±0.003 mM/min. This reaction is favored over the reverse reaction (PEP as substrate) whose kinetics are depicted in Fig 5C; in this case the Km is 0.40±0.09 mM and Vmax is 0.019±0.0004 mM/min. *Sm*Eno enzyme activity was measured over a range of pH values and, as depicted in Fig 5D, this analysis shows that the enzyme’s optimal pH is in the neutral range, with maximal activity at pH 7.5. The enzymes strong preference for Mg^2+^ ions over Ca^2+^ ions is illustrated in Fig 5E; activity is markedly increased by the addition of 1 mM or higher concentrations of Mg^2+^ to the reaction mixture. In contrast, increasing amounts of Ca^2+^ do not increase enzyme activity. Chelating divalent ions by the addition of EDTA reduces r*Sm*Eno activity to trace levels.

Sodium fluoride (NaF) is a known enolase inhibitor and as shown in Fig 5F, NaF can effectively inhibit r*Sm*Eno activity in a dose-dependent manner. At concentrations higher than 6 mM, no enolase activity is detected. Besides NaF, mefloquine (MFQ), a drug used in the prevention and treatment of malaria, has been reported to substantially inhibit enolase activity in *S*. *mansoni* extracts [27]. However, adding MFQ to r*Sm*Eno (at 10, 100 or 500 μM) had only minor inhibitory effect (∼15%) at the highest concentrations tested (Fig 5G, left bars). Schistosomula lysates treated with MFQ also exhibited only minor inhibition of enolase activity (~10%) at the lowest concentration tested but no inhibition in higher concentrations (Fig 5F, right bars).

### *Sm*Eno enhances plasminogen activation

**Fig 6:**
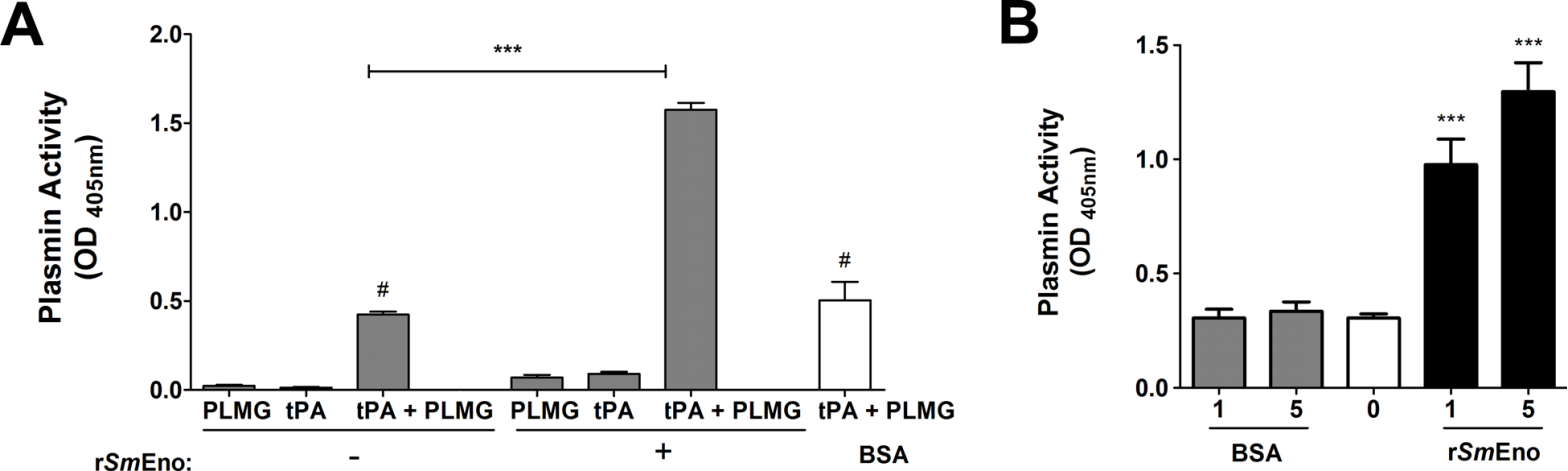
Recombinant *Sm*Eno enhances plasminogen (PLMG) activation. **(A)** Plasmin activity (mean OD_405_ value +/- SD, n = 3) detected in the presence (“+”, right gray bars) or absence (“-”, left gray bars) of r*Sm*Eno. BSA served as negative control, (white bar). tPA is tissue plasminogen activator **(B)** Plasmin activity (mean OD_405_ value +/- SD, n = 3) detected in the presence of increasing concentrations of r*Sm*Eno (black bars) or control protein (BSA) (gray bars) or no protein (white bar). Significant differences from control conditions (reagents themselves without protein) are denoted by ***, p <0.001.

As in earlier similar assays, PLMG or tPA alone (whether in the presence or absence of r*Sm*Eno) has negligible activity in the plasmin generation assay (Fig 6A). While PLMG plus tPA does yield some plasmin (Fig 6A, lane “tPA+PLMG” minus r*Sm*Eno) the addition of r*Sm*Eno dramatically enhances plasmin activation in the presence of tPA in this assay (Fig 6A, lane “tPA+PLMG” plus r*Sm*Eno) (largest gray bar). In contrast the negative control protein BSA has, as expected, no such effect (Fig 6A, white bar). Fig 6B shows that adding more r*Sm*Eno in this assay increases plasmin generation while adding more of a control protein (BSA) does not.

### Suppression of *Sm*Eno gene expression

**Fig 7:**
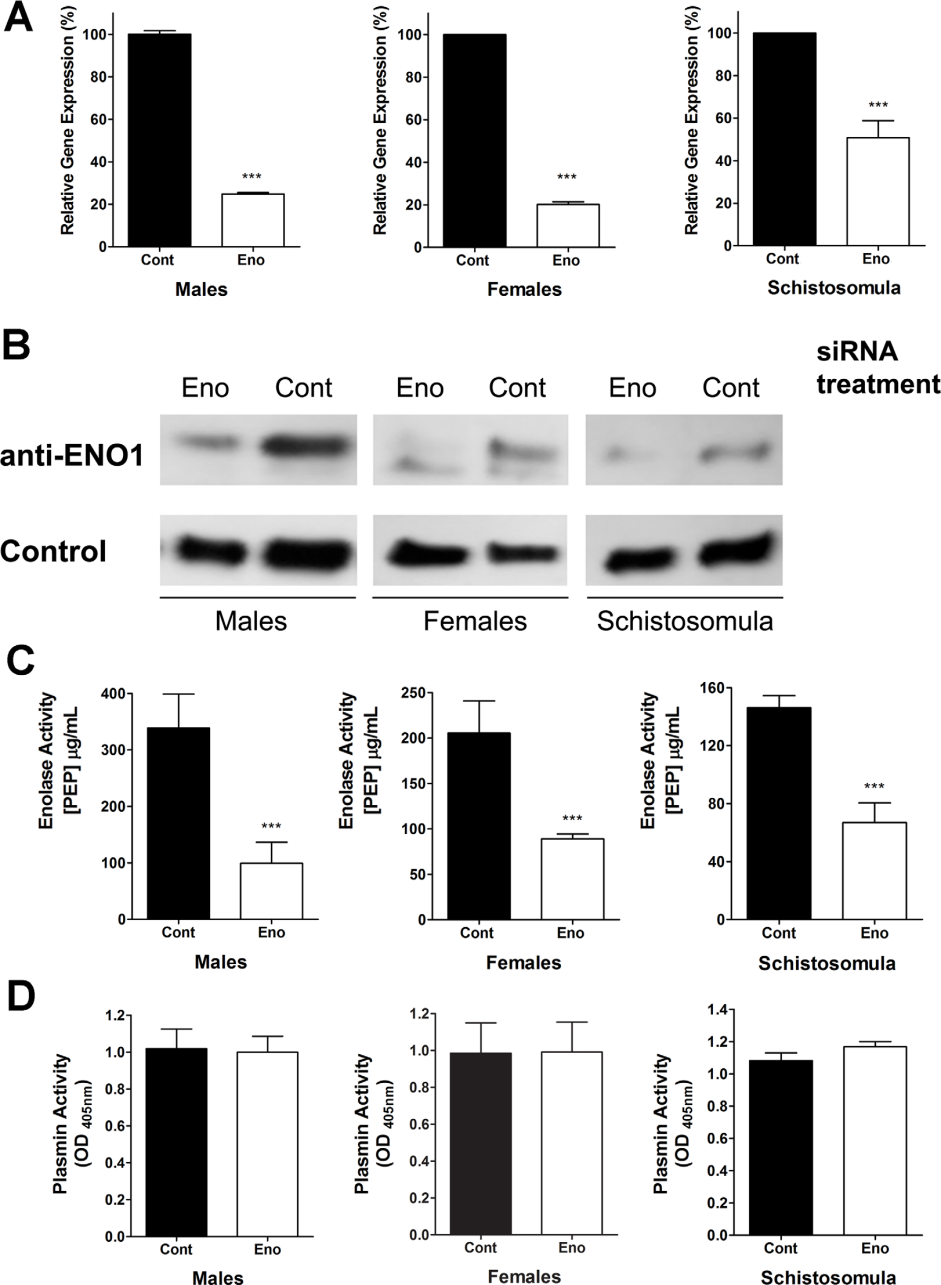
*SmEno* gene suppression using RNA interference. (**A**) Mean level of *SmEno* gene expression (+/-SD, n = 3) in cultured adult schistosome males (left), females (center) or schistosomula (right) at 72 hours after treatment with control, irrelevant siRNA (“Cont” black bars, set at 100%) or siRNA targeting *Sm*Eno (“Eno”, white bars), as determined by qRT-PCR. (**B**) Detection by western blot of *Sm*Eno protein (top row), in extracts prepared from parasites 72 h after treatment with *Sm*Eno (Eno) or control (Cont) siRNAs. Diminished levels of *Sm*Eno protein is seen in the first lane of each group of samples. Western blot analysis detecting a control schistosome protein (lower row) shows roughly equivalent protein amounts per lane. (**C**) Mean (+/-SD, n = 3) surface *Sm*Eno enzyme activity in live adult male (left) or female (center) parasites or schistosomula (right) after treatment with control siRNA (“Cont”, black bars) or siRNA targeting *Sm*Eno (“Eno”, white bars). Significant differences between suppressed compared to control parasites are denoted by ***, p <0.001. (**D**) Plasmin activity (mean OD_405_ value +/- SD, n = 3) detected in live adult male (left) or female (center) parasites or schistosomula (right) after treatment with control siRNA (“Cont”, black bars) or siRNA targeting *Sm*Eno (“Eno”, white bars). All conditions contain plasminogen (PLMG), and tissue plasminogen activator (tPA).

To investigate the importance of *Sm*Eno for schistosomes and to monitor its importance for parasite-driven plasminogen activation, the *Sm*Eno gene was suppressed *in vitro* using RNAi. As shown in Fig 7A, robust gene suppression (ranging from 50–80%) was detected 72 hours after treatment in adult parasites (male and female) and in schistosomula, as measured by qRT-PCR. The suppression of the *SmEno* gene also resulted in a reduction in *Sm*Eno protein production as determined by western blot analysis using anti-ENO1 polyclonal antibodies (Fig 7B). In the case of male (Fig 7B, left panel), female (Fig 7B, center panel) or schistosomula (Fig 7B, right panel), western blot analysis reveals markedly lower *Sm*Eno levels in extracts of parasites treated with siRNA targeting enolase (Eno) compared to parasites treated with a control irrelevant siRNA (Cont). Western blots were stripped and probed with a control antibody to show that all lanes contain roughly equivalent amounts of protein (Fig 7B, lower group).

Next, enolase enzymatic assays were undertaken to determine the levels of enolase substrate (2-PGA) cleavage activity in live, *SmEno*-suppressed parasites versus controls. As shown in Fig 7C, live parasites whose *SmEno* expression was suppressed, unlike controls, had a significantly diminished ability to generate the reaction product PEP from 2-PGA. Reductions in PEP production by the *SmEno*-suppressed groups ranged from 54 to 70%, relative to control parasites (Fig 7C). Next, we compared the ability of live *Sm*Eno-suppressed parasites (males, females or schistosomula) to activate PLMG in the presence of tPA compared to control parasites. Fig 7D shows that robust *Sm*Eno suppression had no measurable impact on the ability of the worms to promote PLMG activation. This is the case for males (Fig 7D, left), females (center) and schistosomula (right).

While *SmEno* RNA levels remained low for at least three weeks post siRNA treatment, suppressed parasites exhibited no significant differences in viability, size, overall morphology or behavior compared to controls.

**Fig 8:**
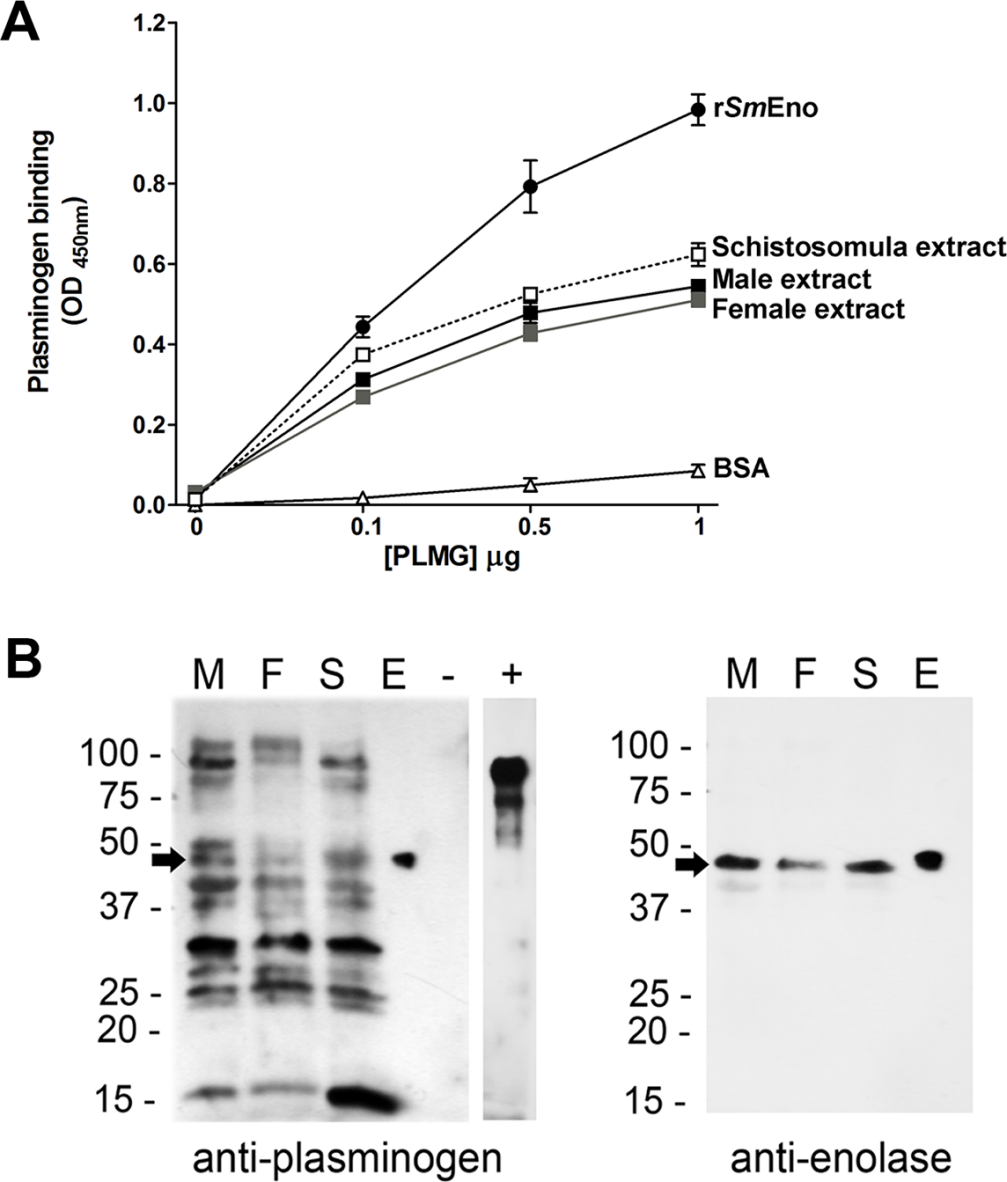
Plasminogen (PLMG) interaction with rSmEno and with schistosome lysates (A) by ELISA or (B) by western blotting. (**A)** ELISA plates were coated with r*Sm*Eno (0.5 μg/well) or the indicated parasite extracts (1.0 μg/well each) and wells were incubated with increasing concentrations of PLMG (0–1 μg) in triplicate. As a negative control, some wells were coated with BSA (0.5 μg/well). Anti-PLMG antibody was used to detect PLMG following standard ELISA conditions. The lines represent the mean absorbance values at OD 450 nm (± SD). (**B**) Detection by western blot of schistosome PLMG-binding proteins (left panel). Lanes contain extracts from males (M), females (F) and schistosomula (S), as well as pure r*Sm*Eno (E), BSA (“-”, negative control) and commercially-obtained PLMG (“+”, a control for anti-PLMG antibody binding). Multiple bands in the schistosome extracts bind PLMG. The arrow indicates the position of migration of *Sm*Eno, here revealed to be a PLMG-binder. No binding to the negative control protein (BSA) is seen. The membrane was stripped and re-probed with anti-ENO1 antibody (right panel). A single, prominent ~47-kDa SmEno band is detected (arrow) in the extracts of males (M), females (F) and schistosomula (S) and in the case of purified rSmEno (E). Images are representative of three replicate experiments.

The PLMG-binding potential of r*Sm*Eno and of total parasite extracts was investigated using two different methodologies: ELISA (Fig 8A) and western blotting (Fig 8B). For ELISA, plates were first coated with r*Sm*Eno and PLMG was then added (at concentrations ranging from 0–1 μg/well) and any bound PLMG was detected using commercially obtained anti-PLMG antibody. The data confirm that r*Sm*Eno (but not the negative control protein BSA) is a PLMG-binding protein. In addition, plates were coated with extracts of male worms, female worms or schistosomula before adding PLMG. This work showed that the extracts contain molecules that bind PLMG and that this binding is dose-dependent (Fig 8A). Little or no difference was detected between the different parasite extracts tested. For western blot analysis, purified r*Sm*Eno as well as lysates of adult male, adult female and schistosomula were resolved by SDS-PAGE and transferred to PDVF membrane. Next the membrane was incubated with commercially obtained PLMG and the presence of any PLMG-binding proteins was detected using anti-PLMG antibody, as described in Methods. Fig 8B (left panel) reveals that parasite extracts contain many proteins capable of binding PLMG under these conditions and these range in molecular mass from 15 kDa to >100 kDa. About 10 major PLMG-binders can be discerned in extracts of male (M), female (F) or schistosomula (S, Fig 8B left panel). Most of the bands are common to all life stages but some differences in the number of bands and the intensity of staining can be seen between stages. Note that r*Sm*Eno binds PLMG in this assay (arrow, lane E, Fig 8B) whereas a negative control protein (BSA, lane “-”, Fig 8B) does not. The “+” lane contains commercially obtained PLMG and here confirms the binding ability of the anti-PLMG antibody used. To identify *Sm*Eno in this blot, the membrane was stripped and re-probed with anti-ENO1 antibody (Fig 8B, right panel). A single, prominent ~47-kDa *Sm*Eno band is detected (arrow) in the extracts of males (M), females (F) and schistosomula (S). Lane E shows anti-ENO1 binding to purified r*Sm*Eno. The detection of a single prominent *Sm*Eno band at ~47 kDa in parasite extracts using anti-ENO1 (as shown in the figure) is important since it establishes the specificity of this antibody.

## Discussion

We show here that intravascular life stages of *Schistosoma mansoni* (adult males, females and schistosomula) can promote the conversion of the zymogen plasminogen (PLMG) to its active form, plasmin, in the presence of tissue plasminogen activator (tPA). In addition to *S*. *mansoni*, the other major schistosome parasites of humans–*S*. *haematobium* and *S*. *japonicum* are also shown to exhibit this ability. This is the first demonstration of this capacity in schistosomes. Plasmin is a serine protease that plays a central role in the degradation of fibrin blood clots. We hypothesize that the ability of intravascular schistosomes to promote plasmin generation as shown here may help them to more freely move and feed in the vasculature unimpeded by the host’s blood clotting apparatus. Any blood clots that begin to form around the worms *in vivo* would likely be efficiently degraded by the parasite’s ability to promote plasmin generation around their bodies. Indeed, *in vivo* the worms do appear to be unperturbed by blood clots [9,28]. We hypothesize that some aspect of the schistosome surface is central to this ability of the worms to activate plasminogen. We focus here on the enzyme enolase which has been detected at the host-interactive *S*. *mansoni* surface by proteomics [29] and which, in other systems, has been shown to bind PLMG and, in some cases, to promote its activation to plasmin [30–34].

Enolase, also known as phosphopyruvate hydratase, is an enzyme that is expressed in a large variety of organisms, from archaebacteria to mammals and is one of the most abundant proteins in the cytosol [16,17]. It catalyzes the dehydration of 2-phospho-D-glycerate (2-PGA) to phosphoenolpyruvate (PEP) in the penultimate step in glycolysis. Besides long being recognized as a cytosolic protein important in sugar metabolism, enolase has more recently been found on the surface of a variety of organisms and cell types [16]. Many pathogens have been shown to express enolase not only in their cytosol but additionally on their surface, [11,12,34–37]. In some schistosome species (*S*. *bovis* and *S*. *japonicum*) enolase has been identified at the host-parasite surface and, in recombinant form, these enolases have been described as PLMG activators [19,20]. In this work we set out to characterize *S*. *mansoni* enolase (here designated *Sm*Eno) and to evaluate if/how it contributes to the parasite’s ability to activate PLMG.

Using tegumental proteomic and genomic information as a guide, we cloned the *Sm*Eno gene, as described in Methods. The *Sm*Eno predicted protein sequence is highly conserved; it exhibits high levels of identity with homologs from other *Schistosoma* species (88–94%), and with human enolase (75% identity). The *Sm*Eno mRNA is one of the few schistosome transcripts that have been shown to be processed by trans-splicing through the addition of a spliced leader RNA [38].

We show here that highest relative expression of *Sm*Eno is seen in the intravascular life stages and particularly in schistosomula. A similar expression pattern was reported for *S*. *japonicum* enolase [20]. Immunolocalization experiments using polyclonal anti-ENO1 antibody show that *Sm*Eno is found throughout the parasites and this is not surprising for an important glycolytic enzyme. In addition, and in accord with *S*. *mansoni* tegumental proteomic analysis [21–23], *Sm*Eno is also clearly detected by immunofluorescence in the schistosome tegument.

An exterior location for *Sm*Eno is confirmed by the fact that live intact parasites all display robust enolase enzymatic activity. Our inability to detect enolase activity in medium in which schistosomes were cultured for up to 48 hours demonstrates that the parasites did not secrete the enzyme nor was it released as a result of damage to the parasites in culture. This is an important point since enolase has recently been shown to be among the most abundant proteins in the schistosome cytosol [39], any damage to the worms could lead to the leakage of the enzyme from internal tissues and confound our results. No such leakage was detected. We find that membrane-impermeable enolase substrate 2-PGA is efficiently converted to PEP by worms in culture. This is the case for all three of the major schistosome species that infect humans. Likewise, all life stages of *S*. *mansoni* tested (males, females and schistosomula) display this activity. Individual males display greater activity compared to individual females and this may reflect their larger size. In keeping with what is known about the biochemical requirements of classical enolases, the activity displayed by the living worms requires Mg^2+^ for maximal activity. Comparing the activity displayed by live schistosomula versus lysates of an equivalent number of schistosomula reveals that the tegumental *Sm*Eno activity accounts for about one fifth of total detectable activity.

*Sm*Eno was expressed as a soluble and functionally active recombinant protein (r*Sm*Eno) in *E*. *coli* and purified to homogeneity by standard immobilized metal affinity chromatography. Enolases catalyze the reversible dehydration of 2-PGA to yield PEP and kinetic analysis shows that *Sm*Eno can likewise perform this function. The enzyme favors the forward reaction (2-PGA → PEP, Km for 2-PGA: 0.26±0.03 mM) versus the reverse (Km for PEP: 0.40±0.09 mM). The enzyme shows optimal activity at near neutral pH (7.5). As described above for the enolase activity displayed by living schistosomes, the activity of the recombinant enzyme also requires Mg^2+^ and activity is enhanced by its addition; Ca^2+^ cannot substitute for Mg^2+^ to promote activity. Removing divalent cations from the assay buffer by the addition of the chelating agent EDTA effectively shuts down the enzyme.

The fluoride ion (F^-^) is known to be an enolase inhibitor since it forms a complex with the essential Mg^2+^ ions and impairs enolase enzymatic function [40]. Here we confirm that r*Sm*Eno is inhibited by NaF at all concentrations tested (0.5-10mM). Enolase activity from *S*. *mansoni* extracts was previously reported to be inhibited by mefloquine (MFQ) in a dose dependent manner. Complete inhibition was seen with 240 μM MFQ [27]. MFQ is best known as an anti-malarial drug that acts by forming toxic complexes with free heme [41]. We find here that there is no dose-dependent inhibitory effect of MFQ on *Sm*Eno activity; at all MFQ concentrations tested (10–500 μM) enolase activity is decreased by no more than 15%. This is the case whether the pure recombinant enzyme is tested or parasite extracts. While our enolase assay measures the generation of PEP directly, the previous MFQ inhibitor study employed a multi-step, indirect measure of enolase action and this, rather than enolase itself, may be perturbed by MFQ [27].

Although we were able to assess enolase enzymatic activity at the *S*. *mansoni* surface, the role this protein plays at this location is likely not related to its ability to interconvert 2-PGA and PEP. We have shown that, just as live schistosomes can activate PLMG (in the presence of tPA), r*Sm*Eno can also fulfill this function. Furthermore, and in keeping with the presence in *Sm*Eno of conserved internal and C-terminal PLMG-binding motifs, we have shown by ELISA and western blotting that r*Sm*Eno can bind to PLMG. In other helminths too, enolase has been identified as a PLMG-binding protein, for instance in the secretory products of *Fasciola hepatica* [42] and *Taenia pisiformis* [31] and in multiple tissues of *Onchocerca volvulus* [43]. As noted earlier, in other schistosomes, enolase binding to PLMG has been shown for *S*. *bovis* [19] and *S*. *japonicum* [20].

We hypothesized the *Sm*Eno–expressed at the host parasite interface and shown here to be able to bind PLMG and enhance its activation–is responsible for the ability of the parasites themselves to likewise bind PLMG and promote activation. To test this hypothesis, *Sm*Eno expression was suppressed using RNAi in *S*. *mansoni* adult males, females and schistosomula and the ability of these parasites to activate PLMG was compared with the ability of non-suppressed controls to activate PLMG. Robust gene suppression was achieved for all life stages tested; *Sm*Eno mRNA levels were significantly lowered in suppressed worms, *Sm*Eno protein levels were diminished as determined by western blotting and the enolase enzyme activity displayed by live suppressed worms was significantly lessened compared to controls. Nonetheless, and surprisingly, this robust *Sm*Eno suppression had no demonstrable impact on the ability of the worms to activate PLMG. The *Sm*Eno suppressed worms activated PLMG to about the same extent as their control counterparts. One explanation for this outcome may be the observation that *S*. *mansoni* extracts contain several PLMG-binding proteins, in addition to *Sm*Eno. *S*. *mansoni* proteins ranging from ~ 15 kDa to >100 kDa were seen to bind PLMG by far-western blotting analysis. Therefore, suppressing the expression of one PLMG-binder–*Sm*Eno–may have little impact if there are several others that can fulfill the same function. Whether the *S*. *mansoni* PLMG-binding proteins seen in this study are expressed at the host-parasite interface where they might access PLMG is not known. Similarly, whether by binding to any of these proteins, PLMG activation is enhanced is likewise unknown. A similar study in *S*. *bovis* reported several (at least 10) tegument proteins as being PLMG-binders, indicating a redundancy in proteins capable of exerting this function [11]. For instance, the tegumental *S*. *bovis* protein annexin (SbANX) has also been shown to bind plasminogen and promote plasmin formation [44]. Despite the abundance of PLMG-binding proteins found in *S*. *bovis*, PLMG binding was observed only to adult male *S*. *bovis* worms and not to adult females [11]. In the case of S. *mansoni*, PLMG binding to extracts of adult females as well as males and schistosomula was observed. Additionally, all life stages examined expressed enolase at the host-exposed surface as determined by immunofluorescence and by enzyme activity assays. Finally all life stages (including females) were able to activate PLMG to generate plasmin, in the presence of tPA. These data indicate a difference in the biology of *S*. *mansoni* versus *S*. *bovis*; in *S bovis* the task of PLMG binding and activation may be confined to the male worms only whereas this is not the case for *S*. *mansoni*.

Finally, it is worth noting that in no instance did *SmEno* suppression lead to a noticeable change in the morphology or behavior of the suppressed worms compared to controls. Since robust *SmEno* gene suppression did not measurably impact the worms, we conclude that high level expression of this protein is not essential for parasite survival in culture. This is perhaps surprising given the known central role of enolase in glycolysis but suggests that the worms in culture can survive even with a (presumably) reduced glycolytic capacity. In contrast, enolase gene knockdown in other helminths (*Ascaris suum* and *Clonorchis sinensis*) increases worm mortality [45,46].

In summary, here we demonstrate that the *SmEno* gene encodes a classical ~47 kDa enolase enzyme that possesses highly conserved active site, Mg^2+^ binding and PLMG-binding motifs. The gene exhibits highest relative expression in schistosome intravascular life stages particularly in schistosomula. In addition to being internal, where the enzyme likely functions in glycolysis, *Sm*Eno is also found in the external tegument of schistosomula and adult worms, where it can bind to and enhance the activation of PLMG. *Sm*Eno catalyzes the reversible dehydration of 2-PGA to yield PEP. It is inhibited by NaF but not MFQ. Knocking-down expression of *SmEno* by RNAi diminishes *Sm*Eno message levels, protein levels and enzymatic activity, but yields no clear phenotype in cultured parasites. In addition, although *Sm*Eno promotes enhancement of PLMG activation to plasmin, *Sm*Eno suppression has no noticeable impact on the ability of suppressed parasites to activate PLMG. *S*. *mansoni* possesses several PLMG-binding proteins in addition to *Sm*Eno which may substitute when *Sm*Eno expression is suppressed. All major schistosome species of humans promote tPA-driven activation of PLMG to generate plasmin. Finally, all *S*. *mansoni* life stages tested exhibit enolase activity on their external tegumental surfaces, as do all three of the major schistosome species of humans. These data suggest that tegumental enolases, like *Sm*Eno play important conserved roles in schistosome-host interaction including being one of several proteins that can drive PLMG activation and control hemostasis around the worms within the blood vessels of their hosts.

## Materials and Methods

### Parasites

Cercariae of *S*. *mansoni* (LE strain) were routinely obtained from infected *Biomphalaria glabrata* snails at the Molecular Helminthology Laboratory at Cummings School of Veterinary Medicine, Tufts University, USA and prepared by exposing infected snails to light for 2 h to induce shedding of parasites. Cercariae numbers and viability were determined using a light microscope prior to infection. Schistosomula were cultured for at least 7 days *in vitro*, as previously described [47]. Adult worms were obtained by perfusion of Swiss Webster mice, 6–7 weeks after infection with approximately 125 (*S*. *mansoni*) or 25 (*S*. *japonicum*) cercariae [47]. Adult *S*. *haematobium* were recovered by perfusion of Golden Syrian hamsters that had been infected with 350 cercariae, 12 weeks previously. Perfusion was performed using RPMI-1640 media or PBS containing 1.5% sodium citrate. Parasites were collected in RPMI medium, counted and immediately cultured in complete DMEM/F12 medium (supplemented with 10% heat-inactivated fetal bovine serum, 200 U/ml penicillin and 200 μg/ml streptomycin, 0.2 μM Triiodo-L-thyronine, 1.0 μM serotonin, and 8 μg/ml human insulin) and were maintained at 37°C, in an atmosphere of 5% CO_2_. Parasite eggs were recovered from the livers of these mice as described [47].

### Chemicals

All reagents were purchased from Sigma-Aldrich, CO (St. Louis, MO, USA) unless otherwise specified.

### Ethics statement

All protocols involving animals were approved by the Institutional Animal Care and Use Committees (IACUC) of Tufts University under protocol G2012-150. All animals were fed, housed and handled in strict agreement with the recommendations of the National Institutes of Health Guide for the Care and Use of Laboratory Animals, the Animal Welfare Act and guidelines established by the American Veterinary Medical Association Panel on Euthanasia.

### Identification and analysis of the schistosome enolase (*SmEno*) gene and predicted protein

Proteomic analysis of the tegument of *S*. *mansoni* revealed the presence there of an enolase homolog which we designate *Sm*Eno [21–23]. Primers complementary to sequence just upstream of the start and just downstream of the end of the predicted *Sm*Eno mature coding sequence were synthesized and used in a PCR with adult cDNA as template. In this manner the entire *Sm*Eno coding region was amplified and subsequently sequenced. The enolase signature sequence and other domains were defined based on ScanProsite online software.

### *SmEno* gene expression analysis

To assess *SmEno* gene expression in schistosome life stages and to monitor gene expression following RNAi treatment, RNA was extracted from the parasites using the TRIzol method (Invitrogen, Carlsbad, USA) following the manufacturer’s instructions. Residual DNA was digested using DNase I (Life Technologies—Carlsbad, USA). cDNA synthesis was performed using 1 μg RNA, an oligo-dT primer and Superscript reverse transcriptase III (Invitrogen, Carlsbad, USA). For quantitative real-time PCR (qRT-PCR), performed using TaqMan Assays, primer sets and reporter probes were customized and reagents were purchased from Life Technologies (Carlsbad, USA). The following primers and probes were used to detect *SmEno* gene expression—primers: *Sm*Eno-F, 5’-GGCCCAAACTAACTTCTTCAACAAA-3’; *Sm*Eno-R, 5’-CGCTTTGGATTGGTAACAGTCAA-3’; and *Sm*Eno probe, 5’-FAM- TCGTCTCCAACAATTTGAA-3’. As an endogenous control, we used the housekeeping triose phosphase isomerase (*TPI*) gene, to compare *SmEno* expression across schistosome life cycle stages. Primers used in this analysis were: *Sm*TPI-F, 5’-CATACTTGGACATTCTGAGCGTAGA-3’; *Sm*TPI-R, 5’-ACCTTCAGCAAGTGCATGTTGA-3’; and *Sm*TPI probe, 5’-FAM-CAATAAGTTCATCAGATTCAC-3’. For relative quantification following gene knock down the *S*. *mansoni* alpha-tubulin gene was used as the control using the following, primers: *Sm*Tub-F, 5’-GGTTGACAACGAGGCCATTTATG-3’; *Sm*Tub-R, 5’-GCAGTAAACCCTTGGTCAGATAATTTTG-3’; and *Sm*Tub probe, 5’-FAM- ATATTTGTCGACGGAAT-3’. Each qRT-PCR reaction was performed using 1 μl of the cDNA, in a final volume of 20 μl. All samples were run in triplicate and underwent 40 amplification cycles on a StepOne Plus system (Life Technologies, Carlsbad, USA). The ΔΔCt method was employed for relative quantification [48]. For graphical representation, ΔΔCt values were normalized to controls and expressed as percentage difference.

### Immunolocalization of *Sm*Eno in *S*. *mansoni* adult worms and schistosomula

To immunolocalize *Sm*Eno, adult worms were first recovered from perfused mice, and schistosomula were prepared *in vitro* as described [49]. Perfused adult worms were embedded in OCT medium (Tissue-Tek, Sakura) and immediately put in liquid N_2_. Five-micrometer cryostat adult worm sections were obtained, allowed to adhere to positively charged glass slides and fixed in acetone for 30 min at −20°C. Cultured schistosomula were fixed in 4% paraformaldehyde for 20 min. Parasites were washed in PBS. Next, schistosomula and parasite sections were blocked with 1% BSA (bovine serum albumin) in PBS (phosphate buffered saline, pH 7.2) (blocking buffer) for 1 hour. The samples were incubated with primary anti-enolase antibody (anti-ENO1; Sigma-Aldrich, Saint Louis, USA) diluted 1:100 for 2 hours. After washing with PBST (phosphate buffered saline, pH 7.2 with 0.05% Tween-20), worms were incubated with anti-rabbit IgG antibody conjugated to Alexa 488 (Molecular Probes, Carlsbad, CA, USA) diluted 1:100 in blocking buffer. Samples were washed, mounted and viewed using an inverted fluorescent microscope (TH4–100; Olympus, Tokyo, Japan) equipped with a Retiga 1300 camera (Q Imaging, BC, Canada). Polyclonal anti-ENO1 antibodies (Sigma-Aldrich) recognize the human enolase sequence ^385^VVGLCTGQIKTGAPCRSERLAKYNQLLRIEEELGSKAKFAGRNFRNPLAK^434^ which is 87% identical to its homologous region in SmEno.

### RNA interference: Preparation and delivery of siRNAs and post treatment analysis

Two gene-specific small inhibitory RNAs (siRNAs) were synthesized commercially (Integrated DNA Technologies, Inc.) and used to induce *SmEno* gene expression knock down. These are siEno1 (5’-CCAUGAGGCUCUUGAGUUACGUGAT-3’, spanning DNA coding positions 128–153) and siEno2 (5’-GCAGAAUCAACCUGGCUUAGUCCTG-3’, spanning DNA coding positions 789–823), both designed with the help of the online IDT RNAi Design Tool (https://www.idtdna.com/Scitools/Applications/RNAi/RNAi.aspx). The siRNAs were delivered to 7-day old schistosomula (~1000) or adult parasites (5–10) by electroporation as previously described [50]. Both siRNAs targeting SmEno led to a comparable degree of target gene suppression. The following, irrelevant, siRNA was used as control: 5’-CUUCCUCUCUUUCUCUCCCUUGUGA-3’. To monitor gene expression at various times post siRNA administration, qRT-PCR was performed using custom TaqMan Assays as described above. To measure protein levels, western blot analysis and enolase enzyme activity assays were performed seven days post siRNA administration as described below. To compare parasite sizes post treatment, images were taken as described for immunolocalization above and the area occupied by individual schistosomula was measured using ImageJ software (U.S. National Institutes of Health, Bethesda, USA). Parasite viability in culture was measured by adding 1 μg/ml Hoechst 33258 to the cultures at room temperature. After 10 min dead parasites (Hoechst positive) were counted microscopically, using a 460 nm reading filter.

### *Sm*Eno protein cloning, expression and purification

A PCR fragment encoding the entire *Sm*Eno coding sequence was obtained using adult worm cDNA as a template and the following primers: *Sm*EnoFw (5’-ACCGGATCCAATGTCCATTTTAACGATCCACGCTCG-3’) and *Sm*EnoRv (5’- AAACTCGAGTTATACTTTGGGATGGCGGAAG-3’). These primers contain, respectively, a *Bam*HI and an *Xho*I restriction site (underlined) to assist subsequent cloning into the expression vector pTrcHisB (Life Technologies, Carlsbad, USA). This cloning strategy generates a poly-histidine (poly-his) tag at the N-terminus of *Sm*Eno. The resulting plasmid (pTrcHisB containing the poly-his/*SmEno* sequence and designated pTrcHisB::*Sm*Eno) was transformed into *Escherichia coli* BL21 Star (DE3) competent cells (Life Technologies, Carlsbad, USA). *E*. *coli* cells harboring the expression plasmid were grown to OD_600_ 0.5, then isopropylthiogalactoside (IPTG) was added to a final concentration of 1 mM to induce gene expression. Four hours later, bacteria were harvested by centrifugation and the cell pellet was resuspended in Bug Buster Lysis buffer (Life Technologies) and recombinant *Sm*Eno protein (r*Sm*Eno) was purified by affinity chromatography on a Ni-NTA Sepharose column following the manufacturer’s instructions (Life Technologies). Fractions containing recombinant *Sm*Eno, identified by SDS/PAGE-12% were dialyzed against PBS pH 7.2 for 16 hours at 4°C. The purified recombinant protein was quantified using a BCA kit (Pierce, Waltham, USA).

### Enolase activity

Classically, enolase enzymes catalyze the dehydration of 2-phospho-D-glycerate (2-PGA) to phosphoenolpyruvate (PEP) in the penultimate step in glycolysis. Schistosome enolase activity was determined by direct monitoring an increase in PEP absorbance at 240 nm at 25°C using a Synergy HT spectrophotometer (Bio-Tek Instruments, Winooski, VT, USA). The forward reaction (2-PGA→PEP) results in an increase in optical density (OD) 240, while the reverse reaction (PEP→2-PGA) results in a decrease in OD_240_. In some cases, a continuous spectrometric assay over 4 h was performed in Costar UV transparent 96-well flat bottom plates (Corning Inc. Corning, NY, USA). For routine enolase activity measurements, the assay (200 μl) contained 20 mM HEPES buffer, pH 7.4, 0.135 M NaCl, 5 mM KCl, 10 mM glucose, 5 mM MgSO_4_ and 2 mM enzyme substrates [2-PGA (for the forward reaction) or PEP (for the reverse reaction)]. Recombinant *Sm*Eno was used at 0.2 μg/assay. To measure enolase activity of live, intact parasites (~1000 schistosomula or individual adult male or female parasites), worms were first briefly washed in assay buffer without substrate and were then incubated in assay buffer. Reactions, in triplicate, were started by the addition of substrate. PEP levels were determined based on a standard curve generated with commercially obtained PEP at 0–500 μM. To prepare parasite extracts, ~1,000 schistosomula or 10 adult worms were harvested, washed briefly three times with PBS, and homogenized on ice in ice-cold PBS + 1% triton X-100 (~50 μl). Protein concentration was measured using a BCA kit (Pierce, Waltham, USA). After parasites (~1000 schistosomula in replicate) had been cultured in medium for either 2 h or 48 h, enolase activity was monitored in this (2h conditioned and 48 h conditioned) media.

### Characterization of r*Sm*Eno

The Michaelis–Menten constant (Km) for the glycolysis substrate 2−PGA was determined from measurements of initial reaction rates in standard assay buffer at concentrations ranging from 0 to 10 mM. In the reverse reaction, the Km for PEP was determined by varying its concentration from 0 to 4 mM. Reactions were initiated by the addition of r*Sm*Eno at a 0.2 μg/assay. Km values were calculated using computerized nonlinear regression analysis of the data fitted to the Michaelis–Menten equation using Graphpad Prism 5.0.

For studies of enolase activation by divalent metal ions, the standard assay buffer was modified by varying the concentration of added divalent ions (CaCl_2_ or MgSO_4_), or by the addition of 5 mM EDTA. Suspected *Sm*Eno inhibitors (NaF and mefloquine) were tested at varying concentrations and pre-incubated with enzyme for 20 min. The reaction was started by the addition of substrate (2 mM 2-PGA). The effect of pH on *Sm*Eno activity was determined in 200 μl enzyme assays using a wide-range buffer (20 mM) system covering the pH range of 5.5–10.0 (MES, pH 5.5–6.5; MOPS, pH 6.5–7.5; HEPES, pH 7.0–8.0, Tris-HCl, pH 7.5–9.0; Trizma, pH 9.0; Glycine-NaOH, pH 9.0–10). The reaction was initiated by the addition of r*Sm*Eno (0.2 μg/assay) diluted in the corresponding buffer. Continuous assays were carried out for 120 min with readings taken every 5 min.

### SDS-PAGE and western blot analysis

Purified recombinant protein and parasite extracts were analyzed on 4–15% polyacrylamide SDS-PAGE gels (BioRad, Hercules, USA) run as previously described [51]. Proteins were transferred to activated PVDF membrane and blocked with TBST (tris-buffered saline pH 7.5, 0.05% Tween 20) containing 5% dry non-fat milk powder. The membrane was then incubated either with a rabbit monoclonal antibody to the 6xHis-tag (GE Healthcare, Pittsburgh, PA, USA), or with polyclonal rabbit anti-ENO1 for *Sm*Eno detection. Following 1-hour incubation at room temperature, membranes were washed and incubated with goat anti-rabbit IgG conjugated to horse radish peroxidase (1:5,000) for 1 hour at room temperature. The blots were developed using ECL Detection Reagents (Amersham Bioscience, Piscataway, USA) according to the manufacturer’s instructions.

### Plasminogen-activation assay

Specific PLMG cleavage leads to the generation of plasmin. The activation of PLMG can be evaluated based on the amidolytic activity of generated plasmin, as previously described [30,37] with some adaptions for the use of live parasites. The assay was performed in 96-well plates. Live parasites (~1000 schistosomula or individual adult male or female parasites) were first briefly washed and then incubated in 100 μl PBS. Next 100 μl human PLMG (1.5 μg) and tissue-PLMG activator (t-PA, 15 ng) in PBS were added. Some wells contained r*Sm*Eno or control protein (BSA) (0.5 μg in 50 μl). Plates were routinely incubated for 1 hour at 37°C to permit plasmin generation. Finally, the synthetic plasmin substrate (D-Valyl-L-Leucyl-L-Lysine 4-nitroanilide dihydrochloride) was added to the reaction mixture (2 μg in 50 μl PBS) and changes in OD_405_ were monitored continuously for up to 4 hours in an ELISA plate reader.

### Plasminogen-binding assay

*Sm*Eno binding to plasminogen was assayed both by ELISA analysis and by western blotting. For the ELISA, 96-well plates were coated with either 0.5 μg/well r*Sm*Eno or 1.0 μg/well parasite (schistosomula or adult male or adult female) extracts in carbonate buffer, pH 9.6, overnight at room temperature. As a negative control, 0.5 μg bovine serum albumin (BSA) was used. Non-specific binding sites were blocked by incubation with 1% BSA in PBS for 2 hours. After washing with PBST, wells were incubated with different concentrations (0–1 μg) of human plasminogen (PLMG) (Aniara Diagnostica, West Chester, OH, USA) for 16 hours at 37°C. After three washes with PBST, wells were incubated with anti-PLMG antibody (Thermo Fisher Scientific, Waltham, USA) at 1:1,000 for 2 hours at room temperature. After another three washes with PBST, all wells were then incubated with 1:1,000 peroxidase-conjugated anti-rabbit IgG for 2 hours at room temperature. Color reaction was induced by the addition of 3,3′,5,5′-Tetramethylbenzidine (TMB) liquid substrate (Sigma-Aldrich). Reaction was stopped by the addition of 50 μl of 2 N HCl. Plates were read at 450 nm in an ELISA plate reader (BioTek).

For western blot analysis, r*Sm*Eno, parasite extracts, BSA (as negative control) and PLMG (as positive control) were resolved on a 4–15% SDS-polyacrylamide gel (BioRad, Hercules, USA) and then blotted to PVDF membrane. Membranes were blocked with 1% BSA in PBS for 2 h at room temperature. Membranes were then washed with PBST and incubated with 25 μg/ml PLMG in PBST for 16 h at 37°C. After three washes with PBST, membranes were incubated with anti-PLMG antibody at 1:1,000 for 2 h at room temperature. Next the membranes were incubated with 1:1,000 peroxidase-conjugated anti-rabbit IgG for 2 h at room temperature and blots were developed using ECL Detection Reagents (Amersham Bioscience, Piscataway, USA) according to the manufacturer’s instructions. Membranes were stripped using Restore Western Blot Stripping buffer (Thermo Fisher Scientific, Waltham, USA) and re-probed with anti-ENO1 antibody as described above, to test for the presence of *Sm*Eno.

### Statistical analysis

For qRT-PCR data, one way analysis of variance (ANOVA) and Tukey as the *post hoc* test was used. *p*-values were considered significant at <0.01. Statistical analyses were performed using GraphPad Prism 5 (La Jolla, CA).
